# Incidentally Discovered Omental Infarction in a Patient With Right Upper Quadrant Abdominal Pain: A Case Report

**DOI:** 10.7759/cureus.106593

**Published:** 2026-04-07

**Authors:** Chenxi Shi, Talyn Smith, Christopher Jurief, Charles Ro

**Affiliations:** 1 College of Osteopathic Medicine, Kansas City University of Medicine and Biosciences, Joplin, USA; 2 Pathology, Mercy Hospital, Joplin, USA; 3 General Surgery, Mercy Hospital, Joplin, USA

**Keywords:** fat necrosis, laproscopy, omental adhesion, omental infarction, right upper quadrant abdominal pain

## Abstract

Omental infarction is a rare and often under-recognized cause of acute abdominal pain. Its clinical presentation varies by location and may mimic other intra-abdominal pathologies. Small infarctions, such as the one described in this case, may not be easily detectable on imaging and can be overlooked without surgical exploration. We present the case of a multiparous woman in her mid-30s who presented to the emergency department with acute-on-chronic epigastric pain. She was taken to the operating room for presumed biliary pathology. Intraoperatively, a twisted segment of omentum adherent to the peritoneum was incidentally identified, and the patient underwent omental resection with adhesiolysis. Histopathologic evaluation revealed fat necrosis suggestive of ischemia secondary to prior torsion. This case highlights the occult nature of small omental infarctions and underscores the importance of considering this entity in the differential diagnosis during the workup of acute abdominal pain.

## Introduction

Acute abdominal pain accounts for up to 10% of emergency room visits annually, and right upper quadrant (RUQ) pain accounts for a smaller subset of these cases [1]. The most common causes of RUQ pain are hepatic and biliary disorders [2]. An acute exacerbation of chronic abdominal pain prompting an emergency room visit suggests the presence of a superimposed acute pathology and warrants further investigation.

Abdominal pain can arise from a wide array of etiologies. Due to the nature of visceral innervation of the abdominal organs, initial gastrointestinal complaints are often vague and poorly localized [3]. Omental infarction is a rare cause of acute abdomen, accounting for less than 1% of all abdominal complaints [4]. The omentum is classically described as a fatty fold that drapes over the intestines and is composed of a rich network of capillaries and lymphatics that support its important role in immune defense [5]. As a highly vascular structure, the omentum is susceptible to infarction and subsequent necrosis. Causes of primary infarction include idiopathic factors, local trauma, malformations, and anatomical variations [6]. Secondary causes of infarction, such as torsion, have an incidence of 0.0016%-0.37% [7].

The omentum occupies a large portion of the abdominal cavity, and the clinical presentation of omental infarction varies depending on the location of the affected tissue. For example, omental infarction may mimic appendicitis or cholecystitis when it occurs in the right lower quadrant and right upper quadrant, respectively [8]. Symptoms may also have a colicky and postprandial, possibly due to vascular congestion or peristaltic movement, thereby resembling biliary disease [9]. In some cases, rebound tenderness and guarding are also present, possibly due to increased tissue tension, mimicking peritonitis [10]. As a result, omental infarction can be difficult to diagnose because it frequently mimics other causes of acute abdominal pain. Although previous cases have identified larger omental infarctions on computed tomography (CT) or ultrasound, these imaging modalities are less sensitive for detecting smaller infarctions and may appear benign or overlooked, such as the one described in this case [11,12].

Here, we present a case of a patient with a history of biliary dyskinesia and chronic right upper quadrant abdominal pain who was incidentally found to have an omental infarction intraoperatively during an elective laparoscopic cholecystectomy, which was not initially identified on preoperative imaging.

## Case presentation

A multiparous woman of normal body mass index (BMI) in her mid-30s initially presented to the emergency department with acute-on-chronic epigastric pain radiating across the upper abdomen. She reported a two-year history of right upper quadrant (RUQ) abdominal pain, with this episode being the most severe. The pain was exacerbated by eating, particularly fatty foods, and was associated with nausea and vomiting. She had no significant past medical and surgical history or trauma to the abdominal wall. Physical examination was notable for epigastric tenderness, and vital signs were within normal limits. Pertinent laboratory studies (Table 1), including lipase, amylase, aspartate aminotransferase (AST), alanine aminotransferase (ALT), alkaline phosphatase, total bilirubin, and white blood cell count, were all within normal limits.

**Table 1:** List of pertinent laboratory studies Pertinent laboratory studies obtained during the initial emergency room visit were within normal range. AST: aspartate aminotransferase, ALT: alanine aminotransferase, HCG: human chorionic gonadotropin

Laboratory test	Patient value	Reference range
White blood cell count	5.9 K/uL	4.3-11.0 K/uL
Bilirubin total	0.3 mg/dL	0-1.2 mg/dL
Lipase	22 U/L	13-60 U/L
Amylase	50 U/L	28-100 U/L
AST	27 U/L	0-35 U/L
ALT	20 U/L	0-35 U/L
Alkaline phosphatase	69 U/L	35-104 U/L
Urine HCG qualitative	Negative	Negative

An abdominal ultrasound was unobtainable at the time of presentation. CT (patient declined intravenous contrast) of the abdomen was interpreted as a normal gallbladder without evidence of acute intra-abdominal pathology. The patient was discharged with symptomatic management and scheduled for an outpatient nuclear medicine hepatobiliary study due to suspected biliary etiology.

**Figure 1:**
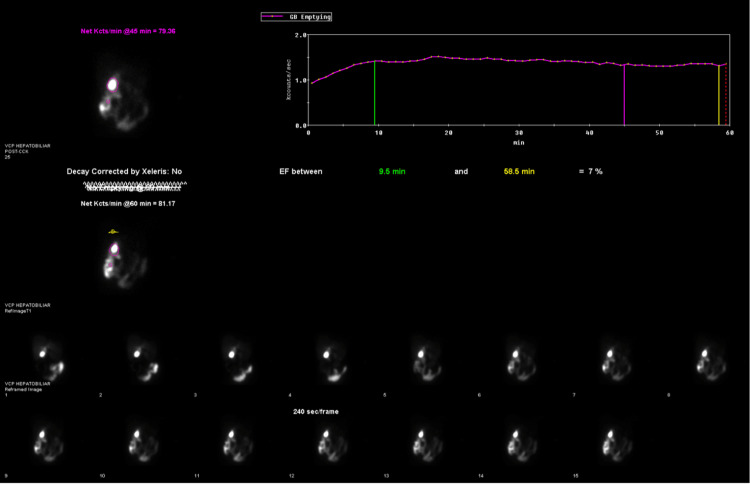
HIDA scan demonstrating a gallbladder ejection fraction of 7%, calculated from tracer activity between 9.5 and 50.5 minutes HIDA: hepatobiliary iminodiacetic acid

A hepatobiliary iminodiacetic acid (HIDA) scan revealed an abnormally low gallbladder ejection fraction of 7% (Figure 1). No gallstones were visualized within the biliary system. The patient was subsequently evaluated by the general surgery service, and an elective laparoscopic cholecystectomy was scheduled for symptomatic biliary dyskinesia.

**Figure 2:**
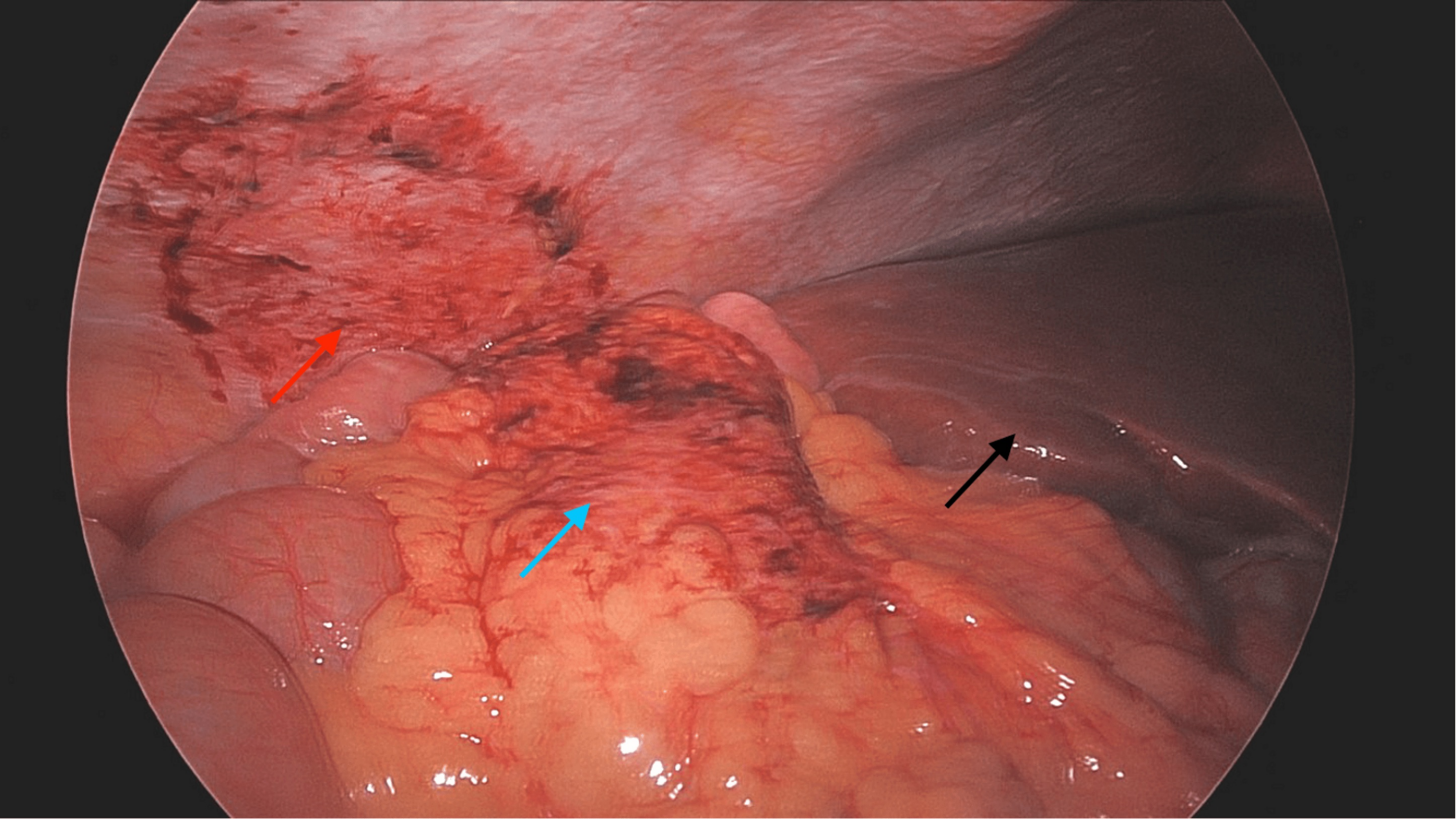
Intraoperative laparoscopic image of the right upper quadrant demonstrating the peritoneal site of prior omental attachment (red arrow) beneath the liver (black arrow), with mobilized omentum (blue arrow)

**Figure 3:**
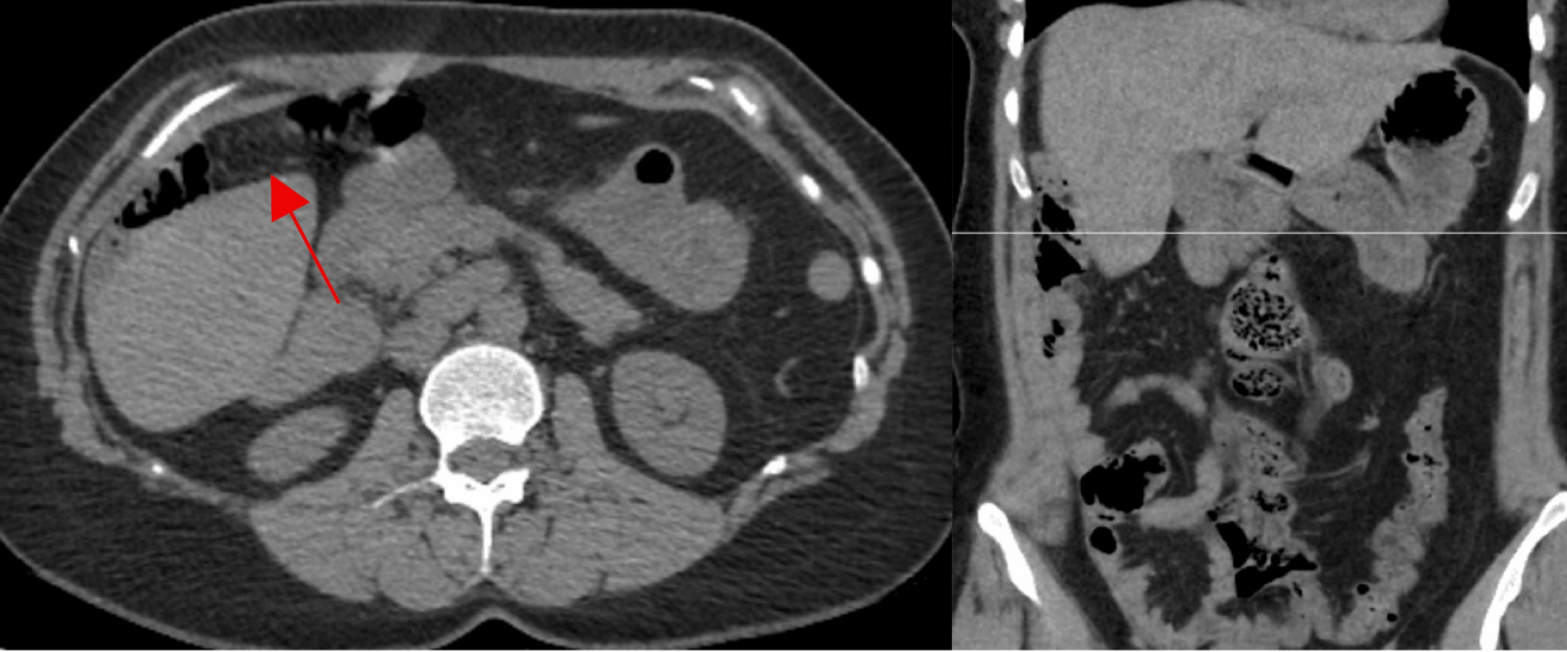
Selected coronal section and corresponding axial section of abdominal and pelvic CT without contrast Postoperative review of the previous scan identified the corresponding site of omental adhesion (red arrow). CT: computed tomography

**Figure 4:**
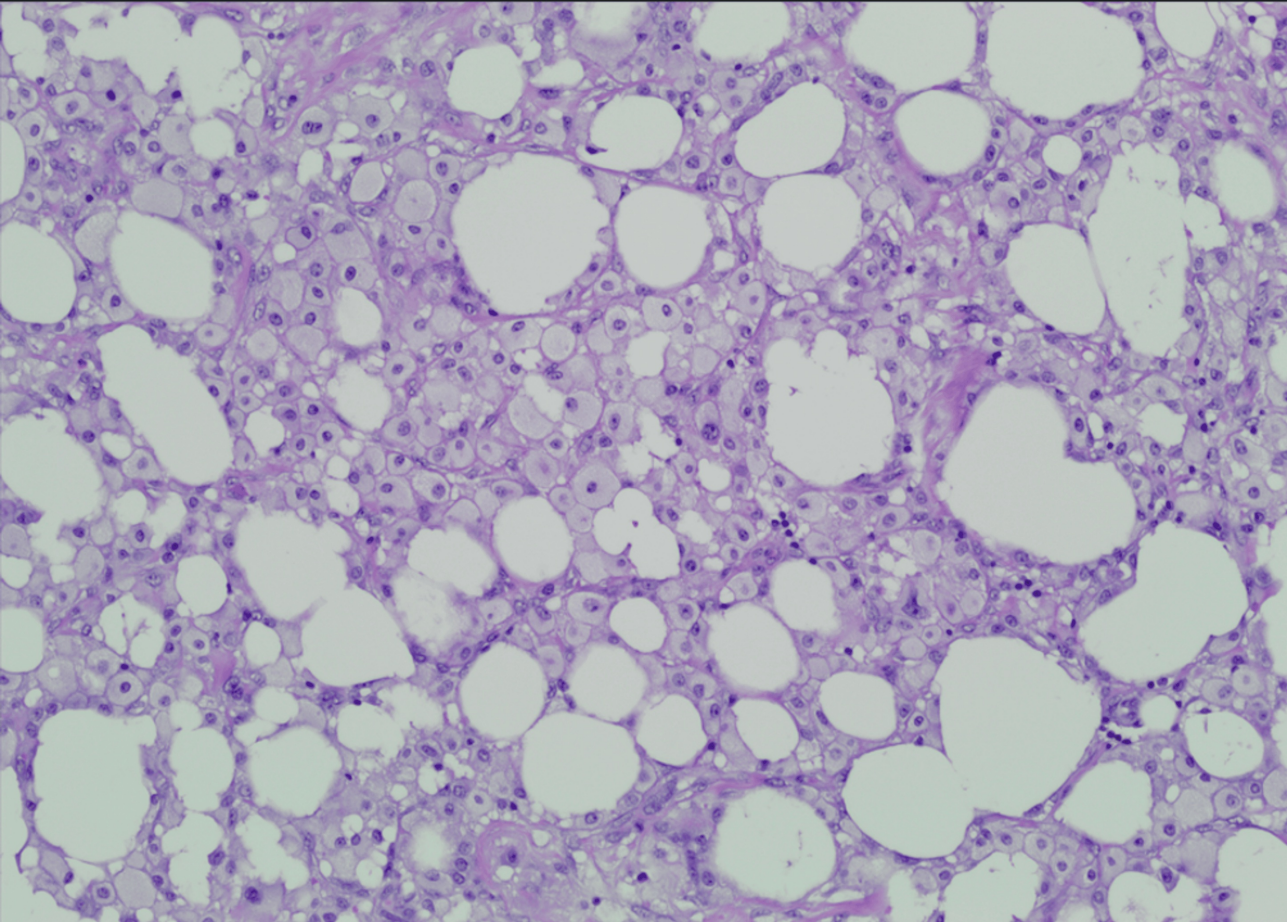
Representative histology of omentum specimen at 10× power There are predominantly mononuclear cells with abundant foamy cytoplasm and cystic spaces. These features are diagnostic of fat necrosis.

Intraoperatively, a small segment of omentum of approximately 4-5 cm was incidentally found to be twisted and adherent to the peritoneal wall inferior to the liver (Figure 2). Lysis of adhesions was performed to allow access to the gallbladder, followed by a partial omentectomy and a laparoscopic cholecystectomy. A retrospective review of the prior CT scan identified the corresponding omental adhesion (Figure 3). Histopathologic examination showed findings consistent with fat necrosis of the omentum (Figure 4) and chronic cholecystitis (image not obtained).

The patient was evaluated two weeks postoperatively and was recovering as expected without residual abdominal pain or additional complaints.

## Discussion

Acute RUQ abdominal pain can arise from a broad spectrum of etiologies. Accurate diagnosis requires a synthesis of epidemiologic factors, symptoms, physical examination findings, and appropriate diagnostic testing. A thorough history, including onset, duration, location, and associated symptoms, combined with a focused physical examination are essential to narrow the differential diagnosis.

Omental infarction is a rare cause of acute abdomen and can mimic other acute abdominal conditions depending on the location of the infarct. In our patient, although she had an underlying biliary disorder for over two years, likely attributable to chronic cholecystitis, her acute exacerbation that prompted emergency department evaluation was likely secondary to a superimposed acute process such as omental infarction.

Small omental infarctions, such as the one described in this case, are difficult to detect on CT due to limited sensitivity and would likely be missed without a laparoscopic evaluation. In this patient, other causes of right upper quadrant abdominal pain were excluded. The abnormal HIDA scan, along with the colicky postprandial nature of her symptoms, was most consistent with biliary dyskinesia, which initially guided the decision for cholecystectomy.

Intraoperatively, a portion of the greater omentum was found adherent to the peritoneum beneath the liver. While abdominal adhesions most commonly result from prior abdominal surgery, this patient had no prior surgical history or trauma, suggesting a congenital etiology [13]. Such adhesions create fixation points that predispose the omentum to torsion and subsequent strangulation. Risk factors for omental torsion include obesity, local trauma, and omental and vascular variations [14]. Other secondary causes of omental infarction may include hernias and other intra-abdominal pathologies [6].

Small omental infarctions can be managed conservatively, as the infarcted fat tissue typically undergoes fat necrosis followed by fibrosis or calcification [15]. However, the timeline of this process is not well defined in the literature, making it difficult to determine the age of the infarction. The management of an intraoperatively discovered omental infarction is dependent on clinical judgement. In this case, the infarction was discovered incidentally during a laparoscopic cholecystectomy, and resection of the infarcted omental segment was elected to prevent potential complications such as recurrent infarction, adhesion, and inflammation. Histopathologic examination of the omentum specimen revealed fat necrosis without calcification, suggesting a relatively recent infarction, which may correlate with the patient’s acute symptom exacerbation that prompted the emergency room visit.

## Conclusions

Omental infarction is an exceedingly rare cause of acute abdominal pain, often presenting with non-specific symptoms that mimic more common intra-abdominal pathologies. Because imaging sensitivity can be limited, clinical judgment and a high index of suspicion are essential for diagnosis and management. In this case, the omental infarction was discovered incidentally during an elective laparoscopic cholecystectomy, and a partial omentectomy was performed to prevent future complications.

## References

[1] Yew KS, George MK, Allred HB (2023). Acute abdominal pain in adults: evaluation and diagnosis. Am Fam Physician.

[2] Revzin MV, Scoutt LM, Garner JG, Moore CL (2017). Right upper quadrant pain: ultrasound first!. J Ultrasound Med.

[3] Gebhart GF, Bielefeldt K (2016). Physiology of visceral pain. Compr Physiol.

[4] Kamaya A, Federle MP, Desser TS (2011). Imaging manifestations of abdominal fat necrosis and its mimics. Radiographics.

[5] Wang AW, Prieto JM, Cauvi DM, Bickler SW, De Maio A (2020). The greater omentum-a vibrant and enigmatic immunologic organ involved in injury and infection resolution. Shock.

[6] LE MJ, JO CG, SP MH, RE EC (1952). Torsion, infarction and hemorrhage of the omentum as a cause of acute abdominal distress. Ann Surg.

[7] Carrillo LM, de Jesús Marín-López J, Díaz-Barrera O, Olvera-Rodríguez JA, Gutiérrez-Gutiérrez LY, Herrera-Gutiérrez J (2023). Omental torsion; an unusual case of acute abdomen. Case report. Int J Surg Case Rep.

[8] Gaba S, Gaba N, Gupta M (2020). Omental infarction imitating acute appendicitis. Cureus.

[9] Paroz A, Halkic N, Pezzetta E, Martinet O (2003). Idiopathic segmental infarction of the greater omentum: a rare cause of acute abdomen. J Gastrointest Surg.

[10] Esposito F, Ferrara D, Schillirò ML, Grillo A, Diplomatico M, Tomà P (2020). “Tethered fat sign”: the sonographic sign of omental infarction. Ultrasound Med Biol.

[11] Alyami HS, Almasaabi SM, Al Swaidan HA, Dhaen H (2023). Omental infarction mimicking acute appendicitis: a case report. Cureus.

[12] Buell KG, Burke-Smith A, Patel V, Watfah J (2017). Omental infarction: the great impersonator. Cureus.

[13] Brüggmann D, Tchartchian G, Wallwiener M, Münstedt K, Tinneberg HR, Hackethal A (2010). Intra-abdominal adhesions: definition, origin, significance in surgical practice, and treatment options. Dtsch Arztebl Int.

[14] Katagiri H, Honjo K, Nasu M, Fujisawa M, Kojima K (2013). Omental infarction due to omental torsion. Case Rep Surg.

[15] Sasmal PK, Tantia O, Patle N, Khanna S (2010). Omental torsion and infarction: a diagnostic dilemma and its laparoscopic management. J Laparoendosc Adv Surg Tech A.

